# Development and Validation of a Novel Ferroptosis-Related LncRNA Signature for Predicting Prognosis and the Immune Landscape Features in Uveal Melanoma

**DOI:** 10.3389/fimmu.2022.922315

**Published:** 2022-06-14

**Authors:** Xiaochen Ma, Sejie Yu, Bin Zhao, Wei Bai, Yubo Cui, Jinglan Ni, Qinghua Lyu, Jun Zhao

**Affiliations:** ^1^ The Second Clinical Medical College, Jinan University, Shenzhen, China; ^2^ Biomedical Research Institute, Shenzhen Peking University-The Hong Kong University of Science and Technology Medical Center, Shenzhen, China; ^3^ Department of Ophthalmology, Shenzhen People’s Hospital, The Second Clinical Medical College of Jinan University & The First Affiliated Hospital of Southern University of Science and Technology, Shenzhen, China

**Keywords:** ferroptosis, lncRNA, immune microenvironment, uveal melanoma, bioinformatics, prognostic value

## Abstract

**Background:**

Ferroptosis is a newly iron-dependent mode of programmed cell death that is involved in a variety of malignancies. But no research has shown a link between ferroptosis-related long non-coding RNAs (FRLs) and uveal melanoma (UM). We aimed to develop a predictive model for UM and explore its potential function in relation to immune cell infiltration.

**Methods:**

Identification of FRLs was performed using the Cancer Genome Atlas (TCGA) and FerrDb databases. To develop a prognostic FRLs signature, univariate Cox regression and least absolute shrinkage and selection operator (LASSO) were used in training cohort. Kaplan-Meier (K-M) and receiver operating characteristic (ROC) curve analyses were used to assess the reliability of the risk model. The immunological functions of FRLs signature were determined using gene set enrichment analysis (GSEA). Immunological cell infiltration and immune treatment were studied using the ESTIMATE, CIBERSORT, and ssGSEA algorithms. Finally, *in vitro* assays were carried out to confirm the biological roles of FRLs with known primer sequences (LINC00963, PPP1R14B.AS1, and ZNF667.AS1).

**Results:**

A five-genes novel FRLs signature was identified. The mean risk score generated by this signature was used to create two risk groups. The high-risk score UM patients had a lower overall survival rate. The area under the curve (AUC) of ROC and K-M analysis further validated the strong prediction capacity of the prognostic signature. Immune cells such as memory CD8 T cells, M1 macrophages, monocytes, and B cells showed a substantial difference between the two groups. GSEA enrichment results showed that the FRLs signature was linked to certain immune pathways. Moreover, UM patients with high-risk scores were highly susceptible to several chemotherapy drugs, such as cisplatin, imatinib, bortezomib, and pazopanib. Finally, the experimental validation confirmed that knockdown of three identified lncRNA (LINC00963, PPP1R14B.AS1, and ZNF667.AS1) suppressed the invasive ability of tumor cells *in vitro*.

**Conclusion:**

The five-FRLs (AC104129.1, AC136475.3, LINC00963, PPP1R14B.AS1, and ZNF667.AS1) signature has effects on clinical survival prediction and selection of immunotherapies for UM patients.

## Introduction

Uveal melanoma (UM) is a rare subtype of melanoma that differs significantly from cutaneous and other types of melanoma in terms of biological and clinical characteristics, with extremely high mortality rates (1, 2). The mean incidence of UM in the USA was five per million (3, 4). Approximately half of UM patients will develop hepatic metastasis (5–8). Although various therapies, such as radiotherapy, local resection, immunotherapy, chemotherapy, and phototherapy, increase the possibility of preserving useful vision, the unsatisfied prognosis of UM has not improved appreciably (9, 10). In addition, recent investigations show that prospective diagnostic tools for UM are rare and limited, indicating the challenge of early diagnosis of UM (11). Therefore, novel predictive models and useful biomarkers for UM patients need to be found and used in clinical practice as soon as possible.

Dysfunction of anti-tumor immunity in tumor microenvironment is a hallmark of melanoma and re-balance of immunosuppressive microenvironment is crucial for melanoma treatment (12). Recently, several immune check-point blockage immunotherapies have been approved for the treatment of melanoma, which contributed to survival of UM patients and provided effective disease control in a fraction of patients (13). Recently clinical practices reported that programmed cell death 1(PD-1) inhibitors only achieved 3.6-4.7% of response rate in patients with UM, and blood biomarkers represented a hopeful mean to evaluate the efficacy of immunotherapy in UM (14, 15). These studies recapped the different characteristics of tumor immune microenvironment of UM and highlighted the importance of developing novel prognostic prediction tools for UM immunotherapy.

Recent years, ferroptosis, as a new category of regulated cell death, is triggered by intracellular iron and it participates in a variety of physiological activities, such as lipid peroxidation and iron metabolism (16–18). The abnormalities of ferroptosis have been associated with the development of hepatocellular, cervical, breast, lung, ovarian, prostate, colorectal, renal carcinomas, and melanoma (17, 19–22). The tumor‐suppressive effect of ferroptosis in carcinomatosis is crucial, including UM (23). Oleic acid prevented melanoma cells from entering ferroptosis, and enhanced lymphatic exposure protected melanoma cells against ferroptosis and boosted their ability to survive (24). Moreover, drugs-induced ferroptosis could potentially enhance anti-tumor immunity response by inhibiting the dedifferentiation of melanoma cells (25, 26). So, evaluation of ferroptosis-related genes (FRGs) or FRLs may provide new clues for diagnoses and prognosis prediction of UM.

Long noncoding RNAs (lncRNAs), as a form of non-coding RNA, play a pivotal role in cellular processes, such as metabolism, neurodegenerative dysfunction, cell cycle regulation, and neoplasia (27–29). LncRNAs are involved in essential carcinogenesis pathways in melanoma, such as the PI3K/Akt, NF-kappa B, and MAPK/ERK (30). Currently, fast-growing studies have explored the ferroptosis-related roles of lncRNAs in tumorigenesis (31). For examples, miR-9 promotes ferroptosis through targeting GOT1 in melanoma cells (32). The miR-137 inhibits necroptosis, and the knockdown of miR-137 boosted anticancer efficacy of erastin by enhancing ferroptosis both *in vitro* and *in vivo*(33). As a result, lncRNA might be examined as a possible target for novel RNA-based anti-UM treatments (34, 35).

Based on the above facts, we hypothesized that FRLs might benefit in the identification of high-risk UM patients and the development of customized treatment plans for them. Herein, we constructed a FRLs signature and confirmed its prognostic value for UM patients. Then, we evaluated the relationship between the immune infiltration landscape and FRLs signature. Finally, we conducted cell experiments to further verify the biological functions of three identified FRLs with known primer sequences.

## Materials and Methods

### Collection and Sorting of Data

The RNA sequencing data in FPKM format and clinicopathological characteristics of UM patients (n=80) were downloaded from TCGA (https://portal.gdc.cancer.gov/). Our research only included UM patients with known survival time and status. Additionally, patients with overall survival of fewer than 30 days in TCGA-UM were excluded to ensure the reliability of the study. The ‘caret’ R package randomly allocated all the selected patients (n=74) into the training and validation cohorts in a 1:1 ratio (36). The ensemble expression matrix was converted into a gene symbol expression matrix using Perl language. Log2 conversion of the data was performed.

### Selection of Potential FRLs

In total, 382 FRGs were obtained from the FerrDb website (http://www.zhounan.org/ferrdb/index.html) database (37). Then, we determined the association between FRGs and lncRNAs by the ‘limma’ R package with Spearman correlation coefficient > 0.6 and *p* < 0.001 as the threshold (38).

### Construction of a Prognostic FRLs Signature

Prognostic FRLs were screened using a univariate Cox regression analysis. Those FRLs with *p*-value < 0.01 were chosen for an overall survival-based LASSO regression analysis to reduce overfit and the number of FRLs by the ‘glmnet’ R package. The risk formula were calculated based on the FRLs expression levels and relevant regression coefficient as follows:


$$\text{Risk score of FR}-\text{lncRNAs signature}=\sum_{\text{i}}\text{Coefficient}\left(\text{FR}-{\text{lncRNAs}}_{\text{i}}\right)*\text{lncRNA Expression}\left(\text{FR}-{\text{lncRNAs}}_{\text{i}}\right)$$

### Evaluation of Risk Model Prediction Ability

The predictive efficacy of the FRLs signature was assessed using K-M and ROC curve analyses by the ‘survival’ and ‘survminer’ R packages. The principal component analysis (PCA) and t-distributed stochastic neighbor embedding (t-SNE) analysis further examined the clustering ability of risk score. We also compared the prediction ability of the FRLs signature with additional clinical characteristics, such as gender, age, and tumor classification.

### Construction of Ferroptosis‐Related LncRNA-mRNA Network

The lncRNA-mRNA coexpression network was created to show the relationship between the FRLs and their related mRNAs using the ‘ggalluvial’ R package and Cytoscape software (version 3.9.0) (39).

### Functional Analysis

Background biological enrichment analyses, such as the Kyoto Encyclopedia of Genes and Genomes (KEGG) pathways and GO biology functions, were examined using GSEA (Version 4.1.0) by ‘ggplot2’ R package. Gene sets items with normal *p* < 0.05 with FDR q < 0.25 were considered significant.

### Comprehensive Analysis in the Immune Infiltration Response, Antitumor Drug Sensitivity, and Tumor Mutation Burden

The difference in immune cell infiltration, the stromal, tumor purity, immune, and ESTIMATE score between the two groups was evaluated using the CIBERSORT and ESTIMATE algorithm (40). The immune function and cell subgroups were investigated using the ssGSEA. In addition, the immune checkpoint and m6A-related genes collected from prior publications were also investigated using ‘limma’ and ‘ggplot2’ R package. The IC50 of frequently used chemotherapeutic medicines was calculated by using R package ‘pRRophetic.’ (41). Tumor Mutation Burden (TMB) was also analyzed using the ‘maftools’ R package.

### Cell Culture and siRNA Transfection

Human highly invasive UM cell line (C918) was purchased from Procell Life Science &Technology, Wuhan, China. C918 cells were cultivated in Roswell Park Memorial Institute medium. Small interfering and negative control RNA were used in knockdown experiments. After 48 hours transfection, cells were collected for RNA extraction or other functional assays. The sequences of siRNA utilized are listed in **Supplementary Table 1**.

### RNA Collection and Quantitative Real Time‐PCR

TRIzol (Invitrogen, Carlsbad, CA) was used to extract total RNA from cell samples. The reverse transcription of the RNA into complementary DNA was subsequently performed using a GoScript reverse transcription system (Promega). Quantitative Real Time‐PCR (qRT‐PCR) validated the transfection efficiency. The list of the primer sequences utilized is shown in **Supplementary Table 2**. The following were the qRT-PCR conditions: initial denaturation takes 5 minutes at 95°C, followed by 40 cycles of denaturation (15 sec at 95°C), annealing (30 sec 59°C), elongation (30 sec at 72°C), and ultimate extension (5 min at 72°C).

### Cell Invasion Assays

Transwell assays were used to assess C918 cell invasion ability. 1.5×10^4^ cells were placed in the top chamber. After a 24-hour incubation period, C918 cells shifted through the membrane were kept for 15 min in methanol and dyed with 10% crystal violet. For invasion assays, Matrigel (Basement Membrane Matrix) was placed into the top chambers 24 h before the trials. The number of C918 cells was counted in ten randomly chosen fields.

### Cell Proliferation

The capacity of C918 cells to proliferate was assessed using the Cell Counting Kit-8 (CCK-8) assay (MCE). After 48 h transfection, C918 cells were seeded at a density of 3500 cells per well in 96-well plates in six repetitions. After 12 h, 24 h, 36 h, and 48 h, the C918 cells were incubated with 10% CCK-8 solution for 1 h at 37°C. A 450 nm wavelength was used to measure the absorbance of living cells.

### Scratch Test

C918 cells were collected and implanted into a six-well plate, where they were grown until 80% fusion. Then the C918 cells were starved in a serum-free medium for 24 hours. The plate was scratched with a one-line design using a 1000μl pipette tip. Scratch healing was examined under the microscope and photographed after a 24-hour culture period.

### Statistical Analysis

The R language software was used to conduct all statistical computations in this research. The Wilcoxon method was used for the two-sample tests. The Kruskal-Wallis test was utilized to assess differences in data among the multiple groups.

## Results

### Acquisition of LncRNA Associated With Ferroptosis

**Figure 1:**
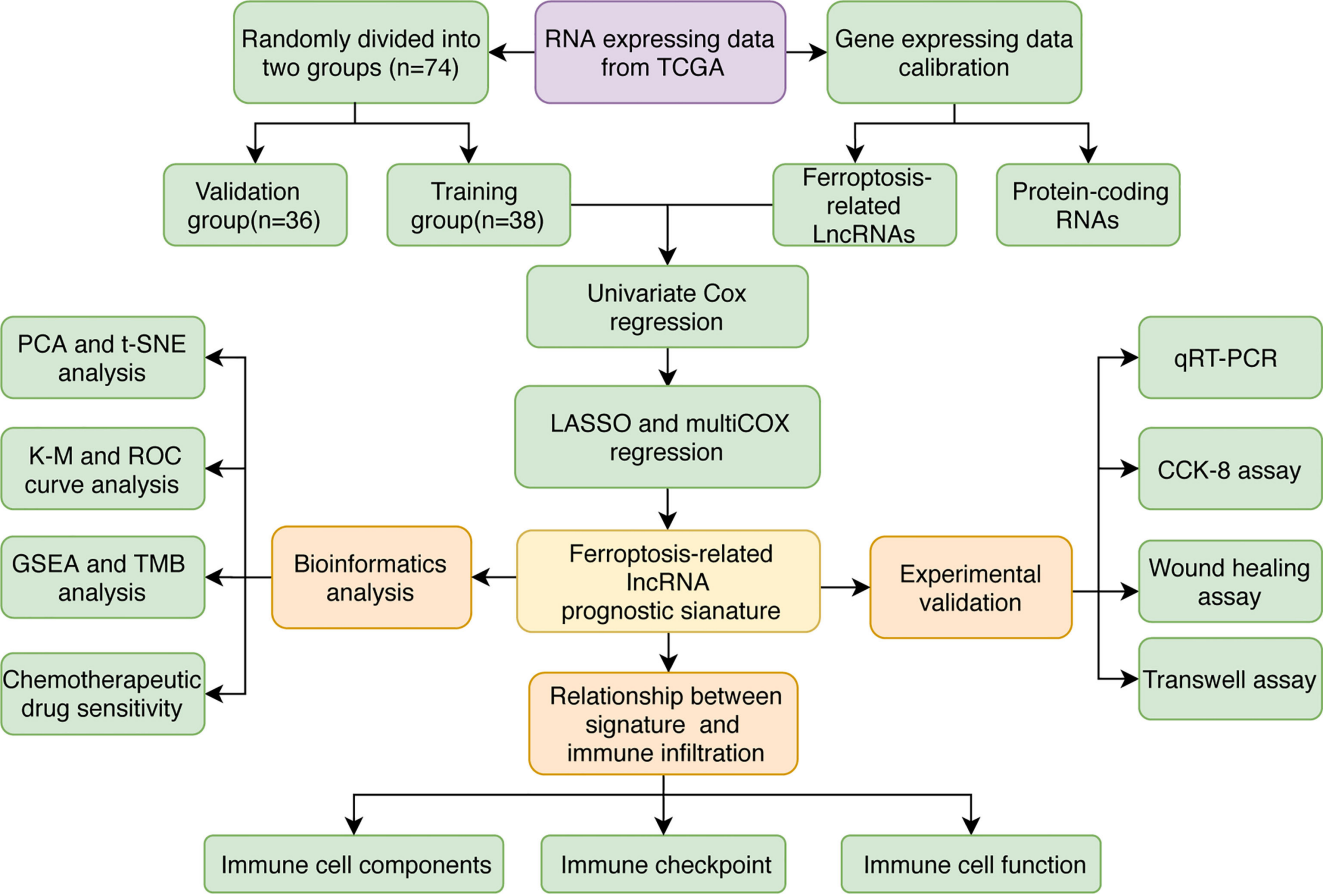
The study flowchart.

The FRLs signature was constructed following the flowchart shown in **Figure 1**. We identified 14,057 lncRNAs and 240 FRGs from the TCGA-UM data. 535 FRLs were identified with the threshold of Pearson correlation coefficients > 0.6 and *p* < 0.001 (**Supplementary Table 3**).

### Development and Validation of Prognostic FRLs Signature

**Figure 2:**
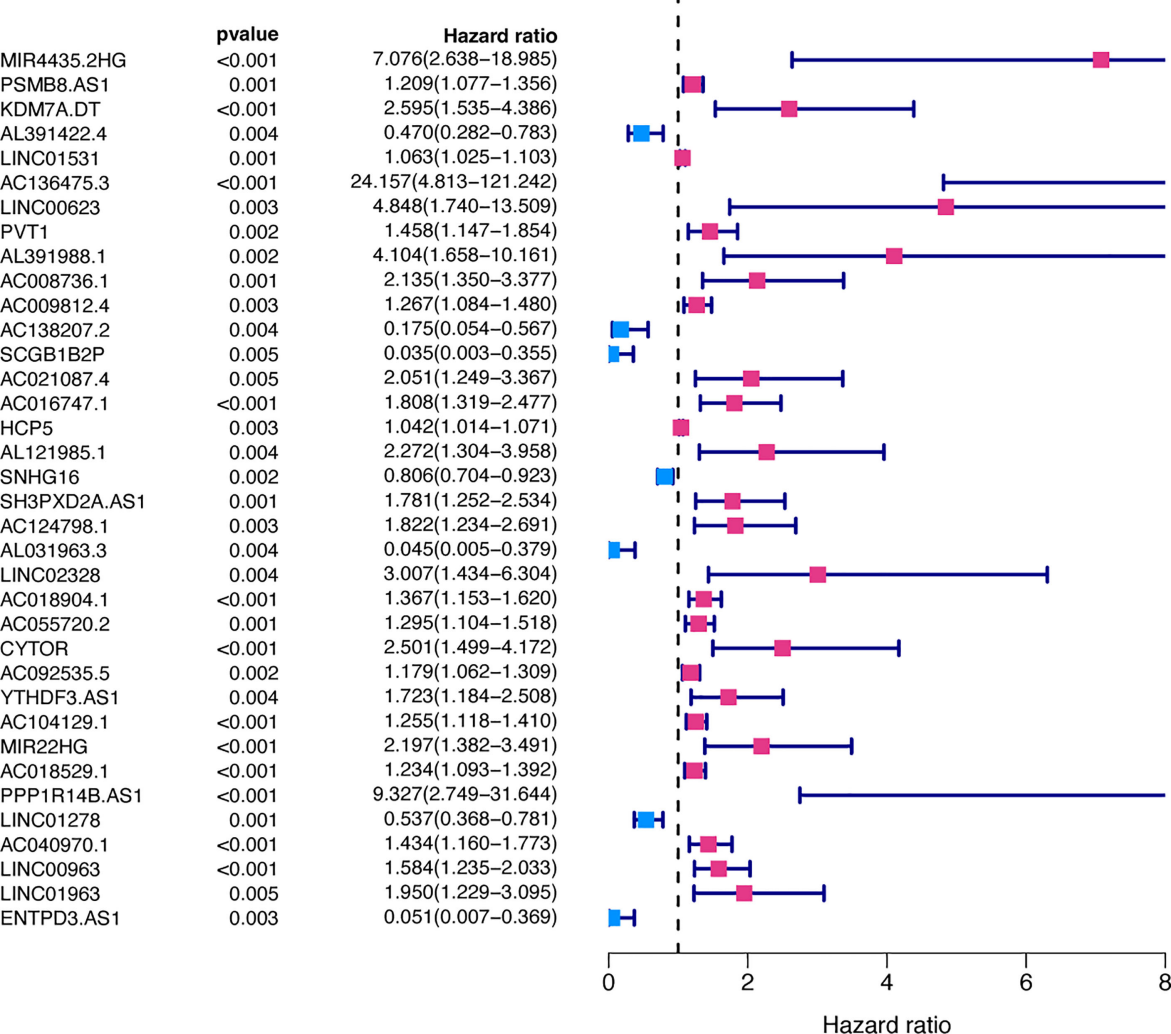
Prognosis-related FRLs from the training set screened using univariate Cox regression analysis.

**Figure 3:**
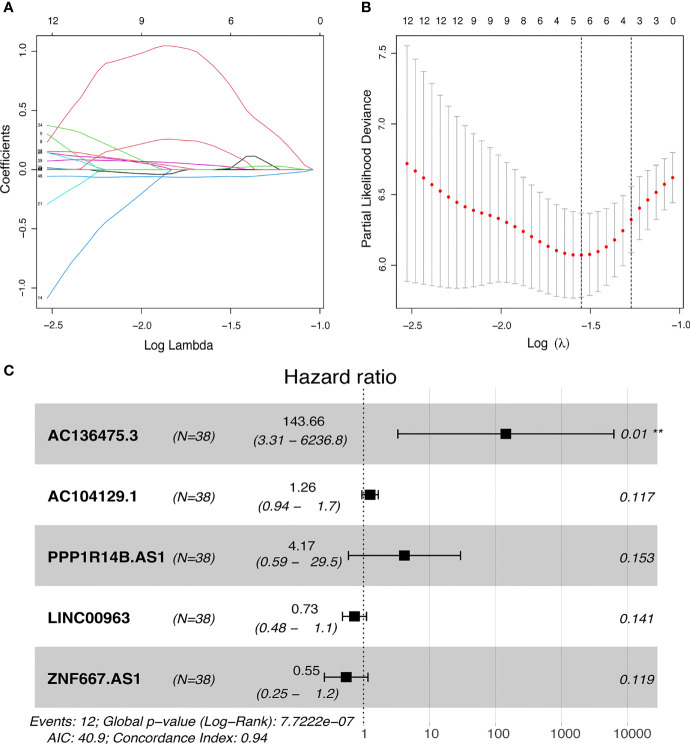
Development of FRLs signature. **(A, B)** LASSO regression model of the prognostic FRLs. **(C)** Forest plot of five FRLs revealed by multivariate Cox regression. **p < 0.01.

49 lncRNAs were uncovered after univariate Cox analysis with *p* < 0.01 filtering (**Figure 2**), five of which were determined using LASSO regression and finally included in the prognostic model (**Figures 3A, B**). The patients were divided into two groups (high-risk and low-risk) according to the median risk score, and the risk score was as follows: risk score = (4.9674 × AC136475.3) + (0.2274 × AC104129.1) + (1.4277 × PPP1R14B.AS1) + (-0.3151 × LINC00963) + (-0.6063 × ZNF667.AS1) (**Figure 3C**).

**Figure 4:**
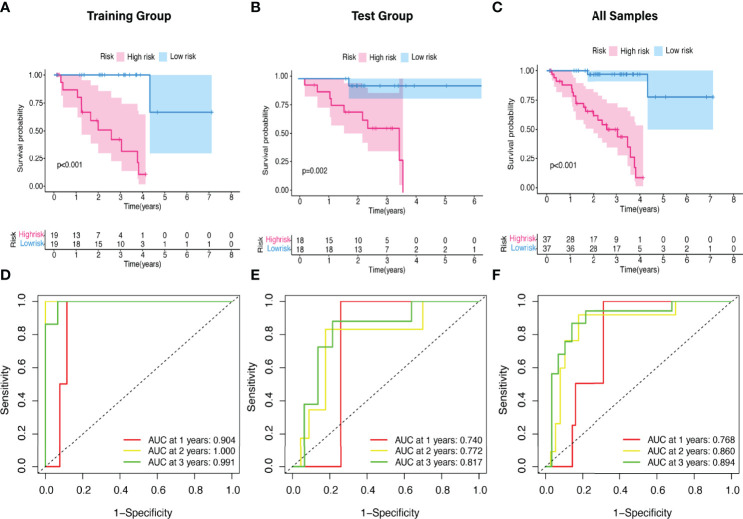
The K-M survival curve and ROC curve of risk score based on five FRLs signature. **(A–C)** Differences in the overall survival of UM patients between the high- and low-risk groups in the **(A)** training, **(B)** test, and **(C)** all cohorts. **(D–F)** ROC curves of risk scores at 1, 2, and 3 years in the **(D)** training, **(E)** test, and **(F)** all sets.

**Figure 5:**
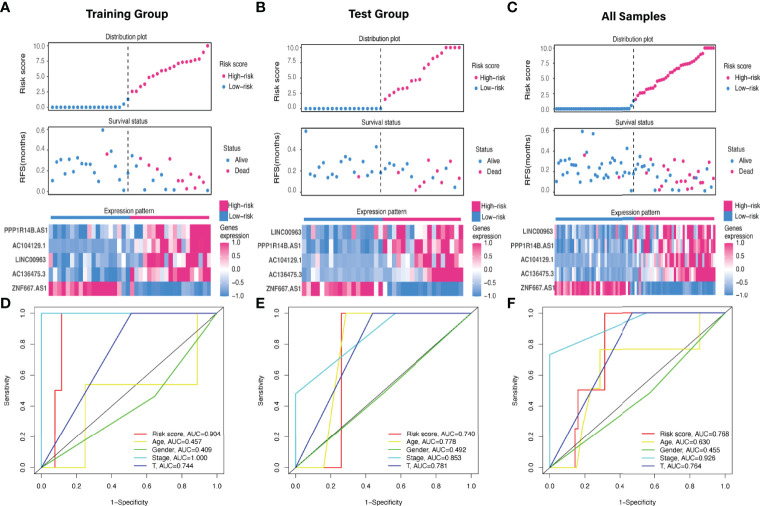
Evaluation of the FRLs signature predictive ability. **(A–C)** The distribution plots, survival status, and expression trends of the different risk score in the **(A)** training, **(B)** test, and **(C)** all cohorts. **(D-F)** ROC curves of risk score, age, gender, stage, and T classification in the **(D)** training, **(E)** testing, and **(F)** all sets.

**Figure 6:**
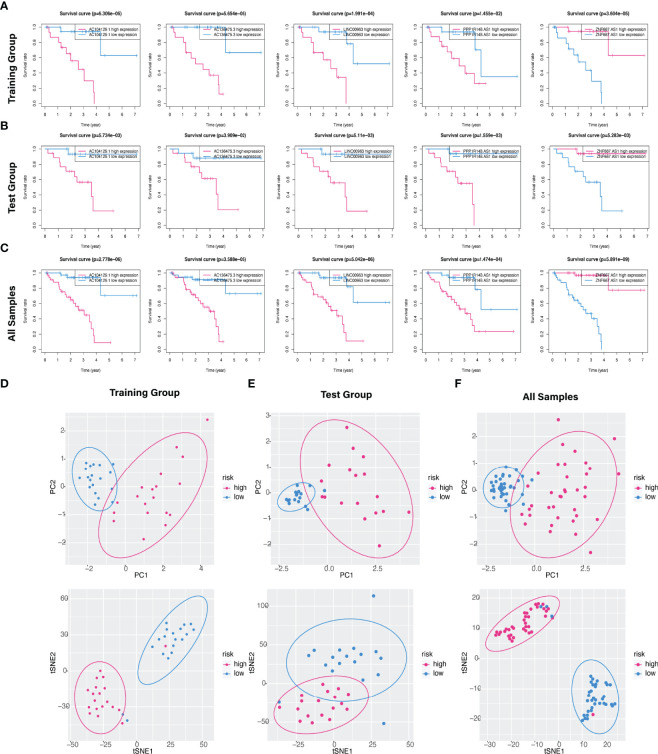
Kaplan–Meier survival analysis of five hub FRLs and discrimination analysis of the risk score. **(A–C)** The K-M survival curve analysis of the five optimal FRLs signature (AC104129.1 AC136475.3, LINC00963, and PPP1R14B.AS1, and ZNF667.AS1) in the **(A)** training, **(B)** test, and **(C)** all cohorts. **(D–F)** PCA and t-SNE diagrams of genome-wide expression profiles of TCGA-UM in the **(D)** training, **(E)** validation, and **(F)** all groups, respectively.

**Figure 7:**
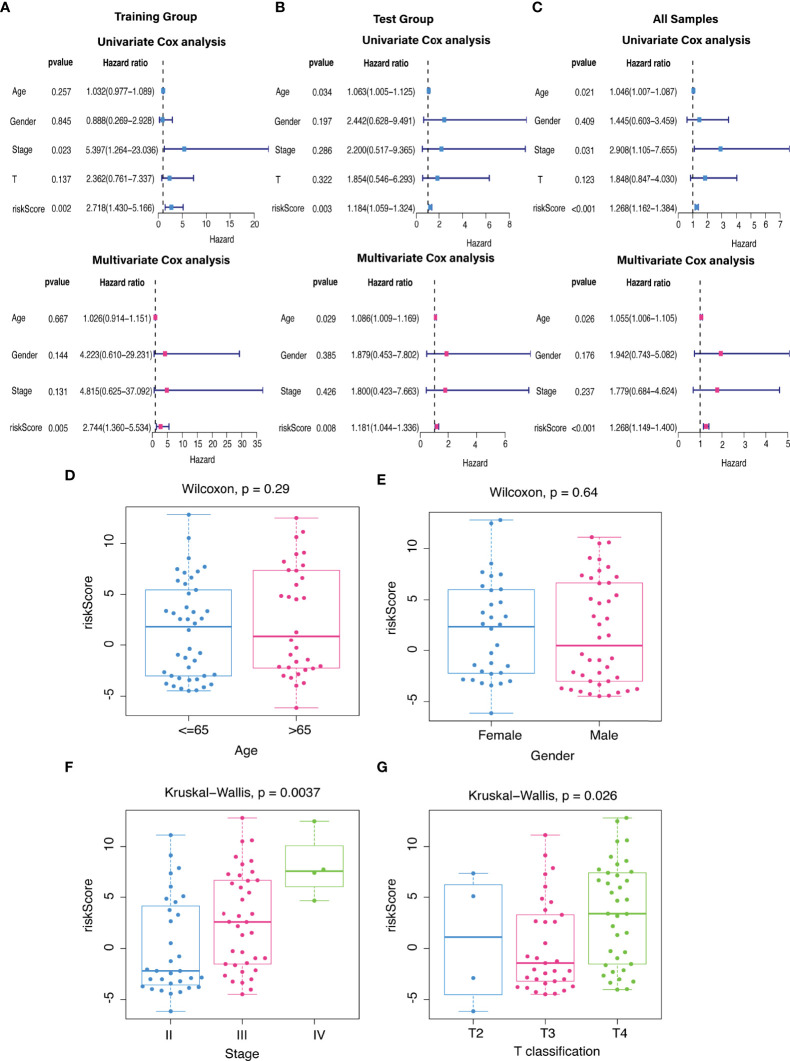
The independent prognostic value. **(A–C)** The forest plots for univariate and multivariate Cox regression analysis in **(A)** training, **(B)** validation, and **(C)** total groups. **(D–G)** Different levels of risk scores in UM patients stratified by **(D)** age, **(E)** gender, **(F)** stage, and **(G)** T classification.

**Figure 8:**
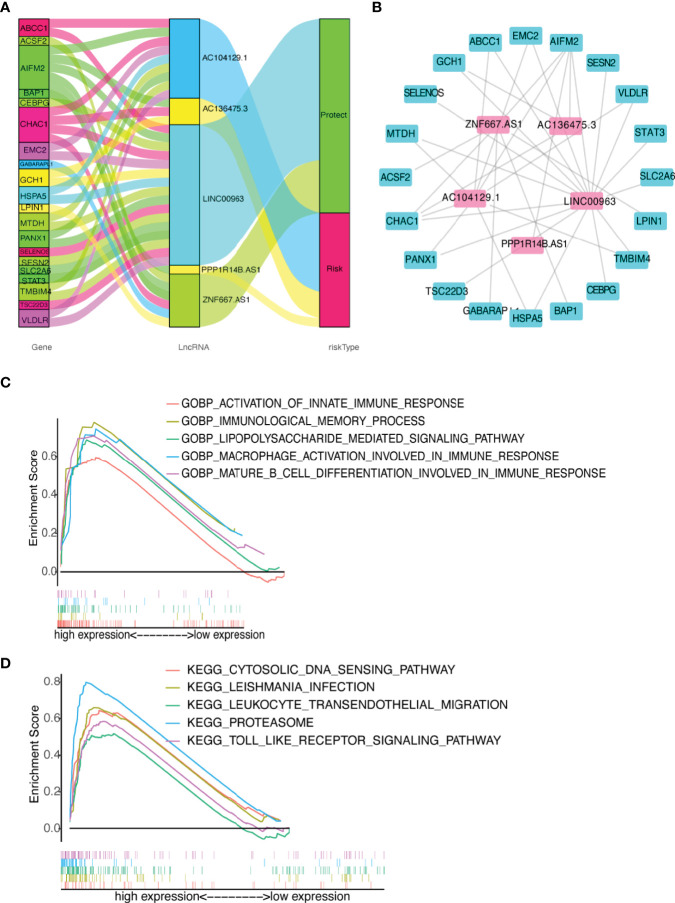
Diagram of lncRNA-mRNA coexpression network and functional analysis. **(A, B)** Alluvial diagram of the relationship between lncRNA and co-expression mRNA with different risk-subgroups. **(C)** GO function annotation of different risk groups. **(D)** The significantly enriched KEGG pathways by GSEA using immune gene set.

The predictive value of the five-FRLs signature was investigated. The K-M curves showed that patients with a high risk score had a considerably increased risk of mortality (**Figures 4A–C**). The areas under the ROC (AUC) values in the training and testing cohorts were 0.904 and 0.740 at 1 year, which showed high accuracy (**Figures 4D–F**). In addition, the survival status, survival times, and expression patterns of patients with UM are shown in **Figures 5A–C**. The ROC findings also revealed that the risk signature was a more relatively precise prognostic indicator than other clinical alternative prediction models for UM (**Figures 5D–F**). Patients with the elevated expression of the discovered FRLs, such as AC104129.1, AC136475.3, LINC00963, and PPP1R14B.AS1, showed a shorter overall life expectancy (*p* < 0.01) (**Figures 6A–C**). The lncRNA distribution of the two groups was shown in the PCA and t-SNE analyses (**Figures 6D-F**), and we obviously judged that the selected UM patients might be better discriminated between the two groups. Furthermore, univariate and multivariate Cox regression analysis showed that only the five FRLs signature could serve as an independent prognostic factor for UM patients (**Figures 7A–C**). Additionally, as the stages and T classification progressed, the risk score increased (*p* < 0.05) (**Figures 7D–G**). This finding implies that the FRL signature is strongly involved in the development and prognosis of UM. Sankey diagram depicted the link between FRLs and FRGs, as well as their risk types in UM (**Figure 8A**). The blue nodes represent genes, whereas the pink nodes indicate FRLs co-expressed with those genes (**Figure 8B**).

### Functional Analysis

Immune-related GO terms and KEGG pathways were analyzed by GSEA. The high-risk group had an accumulation of immune-related GO items (**Figure 8C**), including macrophage activation involved in immune response, immunological memory process, lipopolysaccharide mediated signaling pathway, mature B cell differentiation, and innate immune response. In addition, the KEGG pathways proteasome, cytosolic DNA sensing, Toll-like receptor (TLR), leukocyte transendothelial migration, and Leishmania infection were also enriched in the high-risk group (**Figure 8D**). The details are presented in **Supplementary Table 4**. These results suggested that the newly identified FRLs signature was strongly associated with tumor immune function.

### Differences in the Immune Cell Infiltration, Anti-Tumor Targeted Drug Sensitivity, and Tumor Mutation Burden

**Figure 9:**
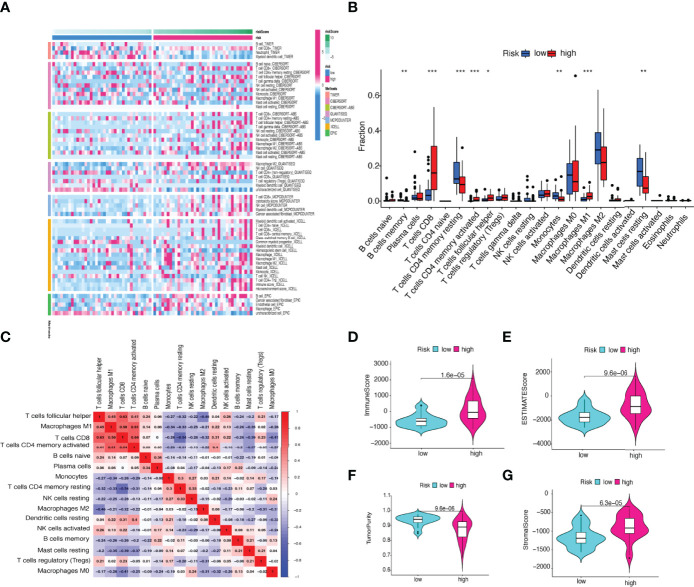
**(A)** Heatmap of immune cell expression using a variety of algorithms. **(B)** The proportion of each typical immune cell between the two groups. **(C)** The correlation of immune cells shown in a heatmap. **(D–G)** Comparison of the **(D)** immune, **(E)** ESTIMATE, **(F)** tumor purity, and **(G)** stromal score. *p < 0.05, **p < 0.01, and ***p < 0.001.

**Figure 10:**
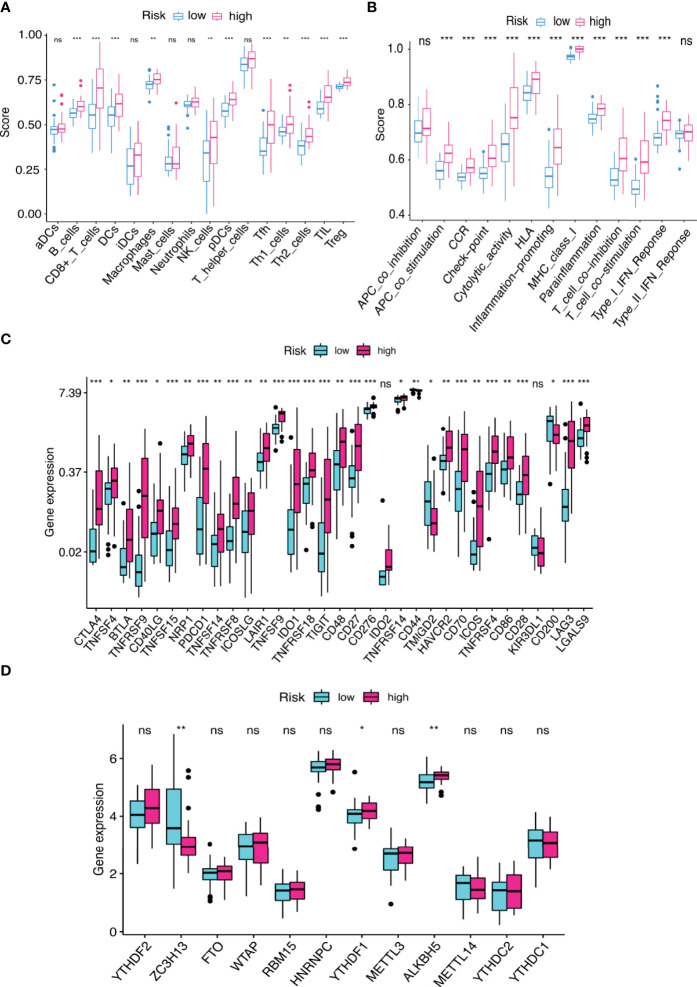
**(A, B)** The score of **(A)** immune cell infiltration proportions and **(B)** immune function by ssGSEA. **(C, D)** The differential gene expression of **(C)** checkpoints and **(D)** m6A between two groups. *p < 0.05, **p < 0.01, and ***p < 0.001. ns, no significance.

We used the CIBERSORT algorithm to examine the immune cell infiltration landscape in UM patients to comprehend more about the association between the FRLs signature and antitumor immune regulation. A heatmap was used to demonstrate the changes in immune cell infiltration between the two groups (**Figure 9A**). **Figure 9B** shows the percentage of each typical immune cell. We found that CD8 T cells, M1 macrophages, memory B cells, CD4 memory T cells, Monocytes, and resting mast cells were significantly different between the two groups. The different immune cell correlation was displayed in **Figure 9C**. **Figures 9D–G** showed that the immune, ESTIMATE, and stromal score were all considerably higher in the high-risk group. Almost all scores of immune cell proportion and immune-related functions differed substantially between the two groups (**Figures 10A, B**). As shown in **Figure 10C**, some validated effective checkpoint immunotherapy targets were overexpressed in the high-risk group, such as PDCD1 (PD-1) and CTLA4. Furthermore, the expression of m6A-related genes YTHDF1 and ALKBH5 was obviously higher in the high-risk group (**Figure 10D**). These revealed that the immune responses of the two groups differed, which might be applied to anti-tumor immunotherapy in UM.

**Figure 11:**
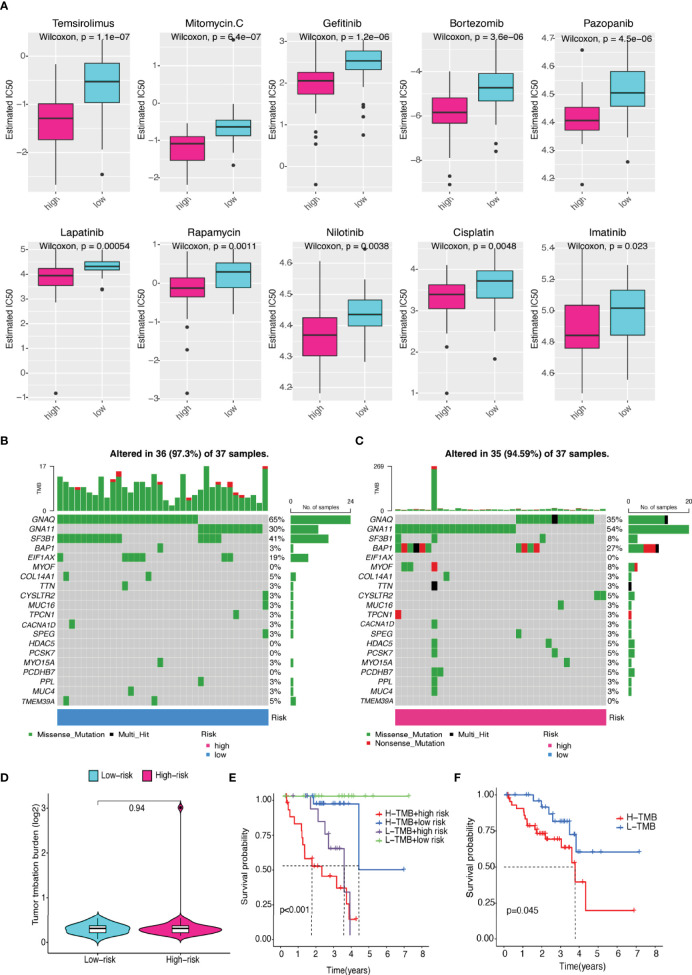
Differences in sensitivity of chemotherapeutic drugs and TMB. **(A)** The IC50 levels of two prognostic risk groups for ten common chemotherapy drugs. **(B, C)** Top 20 gene mutations in **(B)** low- and **(C)** high-risk groups. **(D)** Violin plot of TMB in UM patients between two risk groups. **(E)** The differences in high- and low-TMB levels between high- and low-risk groups in UM patients. **(F)** Kaplan–Meier plot of overall survival for UM patients.

We evaluated the estimated IC50 levels of some chemotherapy medicines between the two groups, and ten typical drugs are shown in **Figure 11A**. The results showed that cisplatin, imatinib, nilotinib, rapamycin, bortezomib, and pazopanib may be potential medications for treating UM patients in the high-risk group.

Although there was no statistically significant difference in TMB levels between the two groups (**Figures 11B–D**), we found that higher TMB had a tendency for lower overall survival rate (**Figures 11E–F**).

### Experimental Validation Analysis

**Figure 12:**
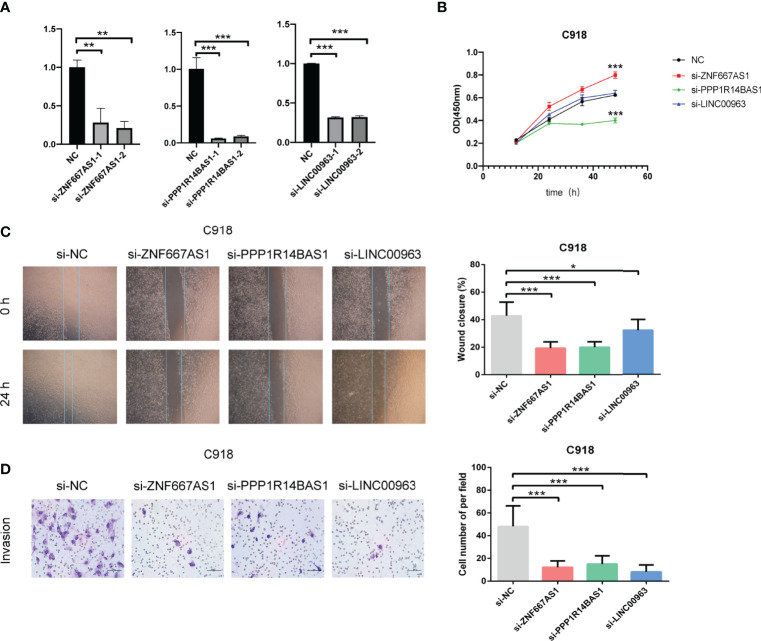
Characteristics of FRLs signature and its functional effect on C918 cells. **(A)** The transfection efficiency verified by qRT-PCR. **(B)** The viability of C918 detected through CCK-8 assay after knockdown of three identified FRLs. **(C)** Cell migration observed under the microscope. **(D)** The differences in the invasive ability of C918 cells. *p < 0.05, **p < 0.01, and ***p < 0.001. “ns” means no significance.

The roles of the five identified FRLs have not been reported in UM. In addition, the detailed primer sequences of AC104129.1 and AC136475.3 are unavailable in gene banks. Thus, to further explore the potential cell function of the other three lncRNAs (LINC00963, PPP1R14B.AS1, and ZNF667.AS1), we used the C918 cell line to construct the lncRNAs knockdown phenotypes. The transfection efficiency was confirmed by qRT–PCR (**Figure 12A**), and both siRNA fragments dramatically reduced the expression of LINC00963, PPP1R14B.AS1, and ZNF667.AS1. Then, we performed a series of assays to study cell function change. CCK-8 assay results suggested that the down-regulation of PPP1R14B.AS1 inhibited the proliferation ability of C918, while the underexpression of ZNF667.AS1 enhanced the proliferation of C918 cells. The knockdown of LINC00963 in the C918 cells could not change the proliferation capacity (**Figure 12B**). Scratch test suggested that after culture for 24 h, scratches of the knock-down group healed slowly and the area of cell migration decreased, indicating that inhibition of LINC00963, PPP1R14B.AS1, and ZNF667.AS1 expression could reduce the migration ability of C918 cells (**Figure 12C**). Furthermore, knockdown of LINC00963, PPP1R14B.AS1, and ZNF667.AS1 attenuated the invasion ability of C918 cells *via* transwell assay (**Figure 12D**). These findings suggest that LINC00963, PPP1R14B.AS1, and ZNF667.AS1 serve as high-risk predictors in C918 cells, and their high expression promotes cancer growth in some way.

## Discussion

Patients with advanced stage UM have a death rate of more than 95% within 5 years (42), so discovering new and effective prognostic biomarkers is crucial for UM. Several studies have recently begun to mine the prognostic lncRNA value in tumors from public databases. For examples, a FRLs signature could be utilized to accurately predict the prognosis of glioma (43). BASP1-AS1 might be used as a biomarker to detect cutaneous malignant melanoma (44). However, to our knowledge, there is a scarcity of studies focusing on FRLs in UM. Many cellular processes, including ferroptosis, are regulated by lncRNAs in experimental studies. Several previously identified lncRNAs are related to shape, TNM stages, diagnosis, and progression of melanoma (45). Therefore, developing a FRLs signature may be useful in predicting UM prognosis and optimizing therapeutic methods.

In this study, we first obtained 240 FRGs and 535 FRLs in TCGA-UM data. Patients with UM were allocated to the training and test groups in a 1:1 ratio. We first identified 49 prognostic FRLs using univariate Cox regression analysis. Then We obtained five FRLs signature using LASSO Cox regression. The following K-M survival analysis showed that UM patients with the high-risk score had a terrible prognosis, but those in the low-risk group had a better life expectancy, demonstrating strong prognostic potential of the newly discovered signature. Additionally, AUC analysis suggested stable performance in different risk-level UM patients. As a result, once the expression levels of the five FRLs are identified, we can forecast the probability of mortality in patients with UM. We also found that risk score was significantly higher in advanced stage (IV) and T classification (IV), indicating that the FRLs signature has a significantly discriminable ability for UM patients. Then, we conducted a series of cell tests to verify the function of these discovered FRLs. These findings suggested that the FRL signature might be exploited as a viable UM prognostic biomarker.

Our FRLs signature included AC104129.1, AC136475.3, LINC00963, PPP1R14B.AS1, and ZNF667.AS1. The roles of the five FRLs have not been reported in UM. This research was the first to utilize the lncRNA AC104129.1 as a biomarker for cancer. Nevertheless, further research on AC104129.1 is needed to understand the deeper mechanisms in the future. The other four lncRNAs have been reported as biomarkers for different carcinoma. For instance, AC136475.3 could be considered as a prognostic factor hepatocellular carcinoma (46). LINC00963 could serve as an oncogene by regulating biological processes, including survival, metastasis, and differentiation (47), was up-regulated in prostate cancer (48), hepatocellular carcinoma (49), osteosarcoma (50), and cutaneous squamous cell carcinoma (51, 52). PPP1R14B-AS1 was identified to be highly expressed in 12 malignancies in the TCGA database. The inhibition of PPP1R14B-AS1 can repress growth and migration in human hepatocellular carcinoma (53). ZNF667-AS1, as a tumor suppressor, could inhibit the viability, migration, and invasion of esophageal squamous cell carcinoma (54). In this study, the CCK-8 experiment revealed that the down-regulation of PPP1R14B.AS1 decreased C918 proliferation. In contrast, C918 cells proliferated more when ZNF667.AS1 was underexpressed. In addition, the knockdown of LINC00963 had no obvious effect on the proliferation of C918 cells. For the scratch migration and transwell assay, the knockdown of the three identified FRLs (LINC00963, PPP1R14B.AS1, and ZNF667.AS1) could suppress migration and invasion in the C918 cell line, similar to the above-mentioned findings.

Additionally, we investigated the correlation between the immune infiltration and the five-FRLs signature. We found that memory B cells, M1 macrophages, activated CD4 memory T cells, Monocytes, CD8 T cells, and resting mast cells were significantly different between the two groups. CD8 T cells have the capacity to promote ferroptosis *in vivo (* 55, 56). In a recent case report, the accumulation of CD8 T cells was detected in hepatic metastases lesions in a patient with UM (57). In our study, the percentage of CD8 T cells was greater in patients with a high risk score. Recent findings show that interferon-gamma produced by CD8 T cells has a tumor-suppressing impact when ferroptosis is activated, suggesting that the immune system may help to prevent carcinogenesis through ferroptosis (55). This could provide fresh insights into the association between ferroptosis and tumor immune microenvironment.

Immune checkpoint inhibitors (including PD-1, CTLA-4, PD-L1, and TIM-3) reduced the activation of immune cell, leading to immunosuppression of the tumor immune microenvironment (58, 59). Anti-PD-1 drugs, such as pembrolizumab, nivolumab, or atezolizumab, were already used to treat individuals with metastatic UM, displaying limited response rates and therapeutic effects in UM patients (14, 60, 61). Anti CTLA-4 drugs, such as tremelimumab and ipilimumab, were also used in patients with metastatic UM. A previous meta-analysis showed that immune checkpoint blockade immunotherapy was helpful for the treatment of UM patients in terms of long-term survival (62). In this study, the PD-1 expression was increased in the high-risk group. CD8 T cells could regulate endocytic recycling of PD-1 and exert synergistic effects with anti-PD-1 therapy in hepatocellular carcinoma (63), and inducing immunogenic ferroptosis in cancer cells also potentiates anti-PD-1 therapy (64), highlighting a promising strategy for cancer immunotherapy.

GSEA results showed that immune system hallmarks, such as immunological memory process, activation of innate immune response, mature B cell differentiation, lipopolysaccharide mediated pathway, and TLR, were considerably enriched in the high-risk score group. TLR is an essential protein molecule in innate immunity and a major regulator of ferroptosis (65–67), and the anti-tumor effectiveness of immunotherapy was aided by enhanced ferroptosis (55). TLR stimulation activated immunoinhibitory signaling pathways such as PD-1 expression (68), which indicates that new targets may be developed to improve therapeutic efficacy for UM.

Predicting the drug sensitivity facilitated in avoiding ineffective use of drugs, revealed new applications for existing drugs, and increased the success rate of therapy (69). Chemotherapy may cause clinical benefits in UM, particularly in patients without bulky liver metastases. The combination of nivolumab and ipilimumab was found to be highly beneficial in metastatic UM (70). Chemotherapy regimens adapted from cutaneous melanoma, such as cisplatin, dacarbazine, and temozolomide, have been utilized in UM patients, and response rates vary between 0% and 15% (71, 72). A triple-drug treatment regimen consisting of cisplatin, vinblastine, and dacarbazine also improved UM patient survival (73). Recent studies have shown that the inducing ferroptosis by blocking STAT3/Nrf2/GPx4 signaling makes osteosarcoma cells more sensitive to cisplatin (74). Our research demonstrated that patients in high-risk group were more susceptible to cisplatin, mitomycin C, gefitinib, bortezomib, pazopanib, lapatinib, rapamycin, and temsirolimus. This may provide novel therapeutic strategies targeting ferroptosis in UM patients.

Admittedly, this study has some limitations. Firstly, the research only covered a limited number of patients owing to the low prevalence of UM, and some deviations might occur. Secondly, the stages N and M of UM patients were excluded because of the unavailable data. Therefore, it is unclear whether they are predictive factors. Lastly, the exact process through which these lncRNAs influence ferroptosis is uncertain. The association between these lncRNAs and FRGs has to be investigated further.

In summary, for the first time, we report and confirm a five-FRLs signature that could be used as a potential biomarker and treatment option for UM. Our results may contribute to predicting the prognosis and developing effective chemotherapy and immunotherapy for UM patients.

## Data Availability Statement

Publicly available datasets were analyzed in this study. This data can be found in TCGA (https://portal.gdc.cancer.gov/projects/TCGA-UVM). Further inquiries can be directed to the corresponding authors.

## Author Contributions

XM, JZ, and QL conceived and designed this study. XM, SY, WB, BZ, YC, JZ, QL, and JN downloaded and analyzed the raw data, performed bioinformatics, and wrote the manuscript. BZ, XM, SY, WB, YC, and JN performed the cell experiments. JZ and QL supervised the research and critically reviewed the paper. All of the writers contributed to the essay and gave their approval to the final version. All authors contributed to the article and approved the submitted version.

## Funding

This study received funding from the Science, Technology and Innovation Commission of Shenzhen Municipality under grant (No. JCYJ20210324113610029 and No. JCYJ20180228164400218).

## Author Disclaimer

The authors’ claims in this article are solely their own, and do not necessarily represent the opinions of their affiliated organizations, the publisher, editors, or reviewers.

## Conflict of Interest

The authors declare that the research was conducted in the absence of any commercial or financial relationships that could be construed as a potential conflict of interest.

## Publisher’s Note

All claims expressed in this article are solely those of the authors and do not necessarily represent those of their affiliated organizations, or those of the publisher, the editors and the reviewers. Any product that may be evaluated in this article, or claim that may be made by its manufacturer, is not guaranteed or endorsed by the publisher.
