# Supplementary material for: Development and Validation of a Novel Ferroptosis-Related LncRNA Signature for Predicting Prognosis and the Immune Landscape Features in Uveal Melanoma
Source: Front Immunol. 2022 Jun 14;13:922315. doi: 10.3389/fimmu.2022.922315 (PMC9238413; doi:10.3389/fimmu.2022.922315)
Supplement: Supplementary file 3 [file Table_1.docx]

The following primers were used in qRT‐PCR:

| ZNF667AS1 |  |  |
| --- | --- | --- |
| forward | (5–GGGAGTGTCCGCCATAAAGT–3) |  |
| reverse | (5–CTACACAAACGCGCGATCAA–3) |  |
| PPP1R14BAS1 |  |  |
| forward | (5–TGCTACCAGGCTTGAACAG–3) |  |
| reverse | (5–CAGGCACAGAGGAAGACAT–3) |  |
| LINC00963 |  |  |
| forward | (5–GGTAAATCGAGGCCCAGAGAT–3) |  |
| reverse | (5–ACGTGGATGACAGCGTGTGA–3) |  |
