# Supplementary material for: Development and Validation of a Novel Ferroptosis-Related LncRNA Signature for Predicting Prognosis and the Immune Landscape Features in Uveal Melanoma
Source: Front Immunol. 2022 Jun 14;13:922315. doi: 10.3389/fimmu.2022.922315 (PMC9238413; doi:10.3389/fimmu.2022.922315)
Supplement: Supplementary file 4 [file Table_2.docx]

| siRNA sequences are as follows: |
| --- |
| Si-ZNF667AS1-1： |
| CCTACTTCTGCATCTTAAA |
| Si-ZNF667AS1-2： |
| CCTTCTCTGCCTGGCTTAT |
| Si-PPP1R14BAS1-1： |
| AGGCTTGAACAGTCTTCAAAT |
| Si-PPP1R14BAS1-2： |
| AGGCTGTAACAAAGATTAAAT |
| Si-LINC00963-1： |
| TTGTACAGTTGGGTAAATCGAGG |
| Si-LINC00963-2： |
| GGCAAGUGCUUUCAACUCU |
